# Drug-induced Fanconi syndrome in patients with kidney allograft transplantation

**DOI:** 10.3389/fimmu.2022.979983

**Published:** 2022-08-19

**Authors:** Zhouqi Tang, Tengfang Li, Helong Dai, Chen Feng, Xubiao Xie, Fenghua Peng, Gongbin Lan, Shaojie Yu, Yu Wang, Chunhua Fang, Manhua Nie, Xiaoqiong Yuan, Xiaotian Tang, Xin Jiang, Xuejing Zhu, Yuxi Fan, Jiawei Peng, Siyu Sun, Mingda Zhong, Hedong Zhang, Longkai Peng

**Affiliations:** ^1^ Department of Kidney Transplantation, The Second Xiangya Hospital of Central South University, Changsha, China; ^2^ Clinical Research Center for Organ Transplantation in Hunan Province, Central South University, Changsha, China; ^3^ Clinical Immunology Center, Central South University, Changsha, China; ^4^ Department of Organ Transplantation, The Fifth Clinical Medical College of Henan University of Chinese Medicine (Zhengzhou Peole’s Hosital), Zhengzhou, China; ^5^ Department of Nephrology, Hunan Key Laboratory of Kidney Disease and Blood Purification, The Second Xiangya Hospital of Central South University, Changsha, China

**Keywords:** Fanconi syndrome, kidney transplantation, adefovir dipivoxil, tacrolimus, renal tubular injury, osteoporosis

## Abstract

**Background:**

Patients after kidney transplantation need to take long-term immunosuppressive and other drugs. Some of these drug side effects are easily confused with the symptoms of Fanconi syndrome, resulting in misdiagnosis and missed diagnosis, and causing serious consequences to patients. Therefore, improving awareness, early diagnosis and treatment of Fanconi syndrome after kidney transplantation is critical.

**Methods:**

This retrospective study analyzed 1728 cases of allogeneic kidney transplant patients admitted to the Second Xiangya Hospital of Central South University from July 2016 to January 2021. Two patients with Fanconi syndrome secondary to drugs, adefovir dipivoxil (ADV) and tacrolimus, were screened. We summarized the diagnostic process, clinical data, and prognosis.

**Results:**

The onset of Fanconi syndrome secondary to ADV after renal transplantation was insidious, and the condition developed after long-term medication (>10 years). It mainly manifested as bone pain, osteomalacia, and scoliosis in the late stage and was accompanied by obvious proximal renal tubular damage (severe hypophosphatemia, hypokalemia, hypocalcemia, hypouricemia, glycosuria, protein urine, acidosis, etc.) and renal function damage (increased creatinine and azotemia). The pathological findings included mitochondrial swelling and deformity in renal tubular epithelial cells. The above symptoms and signs were relieved after drug withdrawal, but the scoliosis was difficult to rectify. Fanconi syndrome secondary to tacrolimus has a single manifestation, increased creatinine, which can be easily confused with tacrolimus nephrotoxicity. However, it is often ineffective to reduce the dose of tacrolomus, and proximal renal failure can be found in the later stage of disease development. There was no abnormality in the bone metabolism index and imageological examination findings. The creatinine level decreased rapidly, the proximal renal tubule function returned to normal, and no severe electrolyte imbalance or urinary component loss occurred when the immunosuppression was changed from tacrolimus to cyclosporine A.

**Conclusions:**

For the first time, drug-induced Fanconi syndrome after kidney transplantation was reported. These results confirmed that the long-term use of ADV or tacrolimus after kidney transplantation may have serious consequences, some of which are irreversible. Greater understanding of Fanconi syndrome after kidney transplantation is necessary in order to avoid incorrect and missed diagnosis.

## Introduction

Kidney transplantation is currently considered as the most effective treatment method for end-stage renal disease. Compared with long-term dialysis treatment, patients who received kidney transplantation have achieved longer survival and better quality of life (1–3).

After kidney transplantation, a large amount of immunosuppressive drugs and other drugs are regularly taken for a long time that can cause drug toxicities, numerous side effects, and complications.

Fanconi syndrome, is a clinical syndrome of a variety of small and medium molecular substance reabsorption disorders caused by the impairment of proximal renal tubular function caused by congenital or acquired factors. It is rare in clinical practice. Fanconi syndrome was first described by Lignac in 1924, and in 1936, Fanconi first characterized children with Fanconi syndrome as having rickets, growth retardation, and diabetes (4–6). The disease has many etiologies, they are divided into primary and secondary, and drug secondary is the main cause.

Transplanted patients with hepatitis B or hepatitis B virus (HBV) carriers need to take long-term medication. The nucleotide analogue adefovir dipivoxil (ADV) is widely used in the treatment of chronic hepatitis B (7, 8). However, a long-term standard dose (10 mg/day) of ADV can cause Fanconi syndrome and secondary hypophosphatemic osteomalacia due to proximal renal tubular dysfunction (9–11). Glucocorticoids also tend to cause osteoporosis, with higher incidence in elderly patients. Therefore, distinguishing osteoporosis in patients taking glucocorticoids and ADV is difficult and thus deserves further research (12).Tacrolimus, one of the most important immunosuppressants, may cause a series of complications after long-term use (13, 14). Tacrolimus can cause acute nephrotoxicity, which clinically manifests as acute oliguria or increased serum creatinine. Afferent glomerular arteriole constriction and direct renal tubular toxicity are the main mechanisms. Acute nephrotoxicity is dose-dependent, which tends to occur early after transplantation (when a certain high dose is taken) and is reversible after dose reduction (15, 16). However, in the late stage after transplantation, whether low-dose tacrolimus taken for more than 10 years can cause kidney damage remains a question.

This study aimed to review and summarize the clinical processes of diagnosis, treatment, and prognosis of Fanconi syndrome secondary to ADV and tacrolimus after kidney transplantation in the Second Xiangya Hospital of Central South University.

## Materials and methods

### Patients

**Figure 1:**
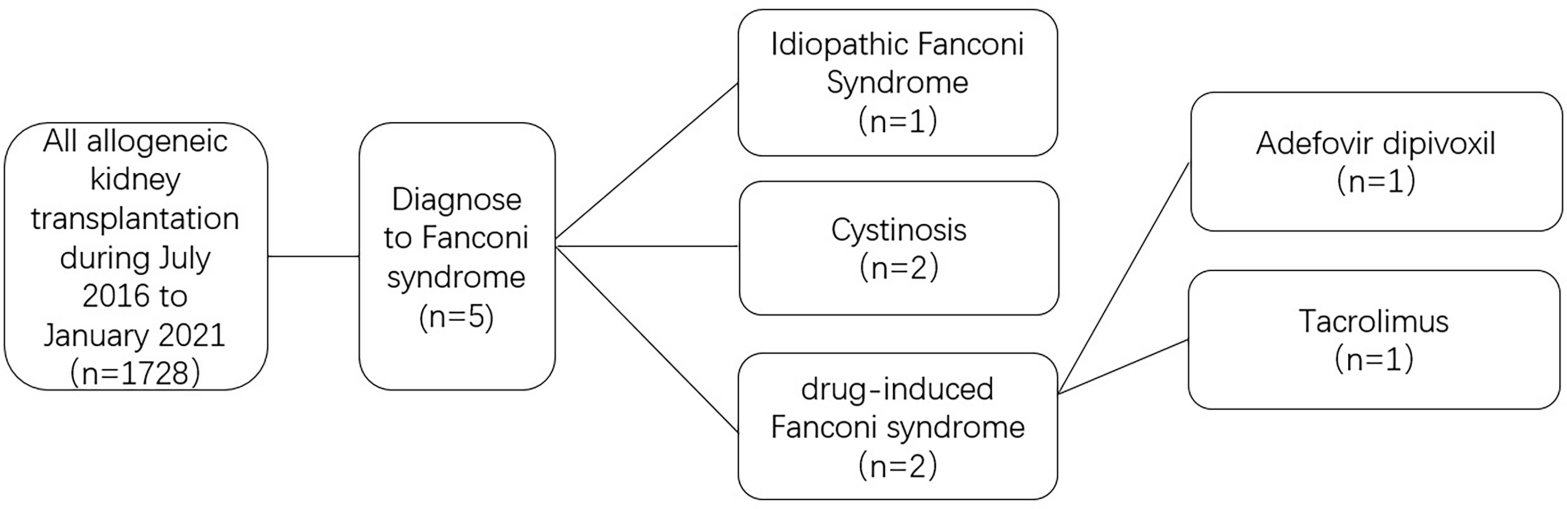
Flow chart of patient selection.

From July 2016 to January 2021, a total of 1728 cases of allogeneic kidney transplantation were performed in the Department of Kidney Transplantation, the Organ Transplantation Center of the Second Xiangya Hospital of Central South University. During this period, five post-renal transplant patients were diagnosed with Fanconi syndrome. The flowchart for the screening of studied patients is presented in **Figure 1**. The diagnostic criteria for drug-induced Fanconi syndrome are the following: a history of taking nucleotides and other drugs; clinical symptoms of obvious bone pain, possibly with decreased muscle strength; proximal renal tubular dysfunction meeting at least two of the following abnormalities: phosphaturia, β2-microglobulinuria, renal glycosuria, renal hypouricemia, and metabolic acidosis; the condition needs to be differentiated from cystinosis, glycogen storage disease, and Dent disease caused by toluene, Lowe syndrome, and proximal renal tubular damage caused by other drugs and poisons. The recovery of renal damage after drug withdrawal also supports the diagnosis. Among the five patients with Fanconi syndrome after renal transplantation, one was diagnosed with idiopathic Fanconi syndrome, two with cystinosis, and two with drug-induced Fanconi syndrome. Among the drug-induced cases, one case was ADV-induced and the other case was tacrolimus-induced, all patients met the diagnostic criteria for Fanconi syndrome.

### Immunosuppressants

All recipients were routinely given methylprednisolone intravenous infusion and oral mycophenolate mofetil before kidney transplantation. Regarding the induction therapy, deceased-donor (DD) kidney transplant recipients were given anti-thymocyte globulin induction before surgery, and living-donor (LD) kidney transplant recipients were given baliximab before surgery. All recipients were treated with triple immunosuppressive therapy after surgery, including tacrolimus [oral, 0.1 mg/(kg d), twice a day], mycophenolate mofetil (oral, 0.75 g, twice a day), and methylprednisolone (the starting dose was 64 mg/day, which was decreased by 8 mg, finally reaching 8–16 mg/day for maintenance). The tacrolimus concentration was measured regularly and maintained at 6–9 ng/mL within 1 year post-renal transplant patients and no less than 6 ng/mL thereafter. Once the diagnosis of tacrolimus-induced Fanconi syndrome was confirmed, tacrolimus was immediately replaced with 3 mg/(kg d) cyclosporin A. The plasma concentration of cyclosporin A was monitored with a valley concentration target concentration of 80–120 ng/mL and a peak concentration target concentration >400 ng/mL.

### Postoperative infection prevention measurements

All kidney transplant recipients were routinely treated with antibiotics to prevent infection, and DD recipients were routinely treated with a “big siege” infection prevention program (anti-Gram-negative bacteria in conjunction with anti-Gram-positive bacteria and caspofungin) for 10 days to prevent infection. Donor kidney lavage fluid, wound drainage fluid, and urine were collected for culture. The anti-infection regimen was adjusted according to the negative culture results. LD recipients routinely used second-generation cephalosporins for infection prevention after surgery, and quinolones were used to prevent infection for 1 week in case of allergy. If the renal function recovered well within half a month after the operation, compound sulfamethoxazole was added to prevent Pneumocystis jirovecii pneumonia(PJP) up to 9 months after the operation. If there was no obvious bone marrow suppression such as leukopenia within half a month after the operation, ganciclovir/valganciclovir was added to prevent cytomegalovirus(CMV) infection until 9 months after the operation. If the patient was positive for hepatitis B surface antigen (HBsAg), intravenous hepatitis B immunoglobulin was administered for 5 days after the operation, followed by long-term oral nucleotide drug anti-HBV treatment and regular monitoring of HBV-DNA level. The patient was treated with long-term oral ADV against HBV due to positive HBsAg after operation.

### Measurements

Retrospective analysis was used to observe and evaluate the general information of patients, including the primary disease before transplantation, type of dialysis, duration of dialysis, type of kidney donation, number of human leukocyte antigen (HLA) mismatches, whether or not it was the primary transplant, the time of Fanconi symptoms, main symptoms and conditions, time from symptom discovery to diagnosis, type and dosage of causative drugs, and duration of medication. Comparative statistical analysis was performed after kidney transplantation from the time of increased creatinine level, bone pain, and osteomalacia symptoms to the day of diagnosis of Fanconi syndrome. Laboratory tests related to Fanconi syndrome, including blood creatinine, blood urea nitrogen, blood uric acid, blood bicarbonate, blood phosphorus, blood potassium, blood calcium, blood glucose, urine β-2 microglobulin, urinary phosphorus, urinary potassium, urinary calcium, urinary glucose, and estimated glomerular filtration rate (eGFR) value, were performed at 2, 7, 18, 500 and 1825 days after diagnosis of Fanconi syndrome. Laboratory tests of bone metabolism and molecular markers included 25 hydroxy-vitamin D (25-OH-VitD), N-terminal mid-fragment (N-MID), procollagen type 1 N-terminal peptide (P1NP), β-isomerized cross-linked C-telopeptide of type 1 collagen (βCTX), alkaline phosphatase (ALP), and intact parathormone (iPTH). Dual-energy x-ray absorptiometry was performed to determine the bone mineral density (g/cm^2^) at the posterior anterior position of the left proximal femoral neck and lumbar spines L1-L4 (bone mineral density lumbar spine, T-in lumbar spine, bone mineral density femoral neck, and T-in femoral neck). Whole-spine splicing X-ray anterior and lateral views, whole-body single-photon emission computed tomography bone scan, and transplanted kidney biopsy pathological examination were performed, and other clinical data were collected.

## Results

### Patient characteristics

The demographic and clinical data of the ADV and tacrolimus patients are presented in **Table 1**. The two patients were both female. The ADV patient was 40 years old when she underwent transplantation (weight, 45.5 kg; body mass index [BMI], 17.7). The tacrolimus patient was 39 years old when she underwent transplantation (weight, 48.7 kg; BMI, 19.7). The primary diseases of the kidneys were chronic nephritis and renal insufficiency, and hemodialysis were performed for both of them before the operation. The durations of dialysis were 7 and 3 months in the ADV and tacrolimus patients, respectively. The donor of the ADV patient was a donation-after-cardiac-death donor, whereas that of the tacrolimus patient was an LD donor (her mother). The HLA mismatch numbers of the ADV and tacrolimus patient were 2 and 1, respectively. Both were undergoing their first transplantation. The kidney transplant operation time of the ADV patient was August 2005, and the Fanconi-related symptoms appeared in July 2016. The main symptoms were bone pain and a series of osteomalacia manifestations such as scoliosis. Fanconi syndrome was diagnosed in July 2017, but before then, related treatment was carried out because it was misdiagnosed as osteoporosis. The diagnosis time was as long as 1 year. The patient was taking ADV (10 mg, once a day) for 8 years after kidney transplantation, and Fanconi syndrome was diagnosed 12 years after the operation. The kidney transplant operation time of the tacrolimus patient was June 2011, and the Fanconi-related symptoms appeared in June 2021. The main symptoms were a series of renal damage manifestations such as increased creatinine. Fanconi syndrome was diagnosed, and intervention was performed. The patient was taking tacrolimus (1 mg, twice a day) for 10 years after kidney transplantation and was diagnosed with Fanconi syndrome 10 years after surgery.

**Table 1:** Demographic and Clinic.

	Patient	
	Patient Adefovir	Patient Tacrolimus
Background	Farmer	Farmer
Gender	Female	Female
Recipient Age,years	40	39
Weight,kg	45.5	48.7
BMI,kg/m^2^	17.7	19.7
Primary Renal Disease	chronic nephritis	chronic nephritis
Dialysis type	haemodialysis	haemodialysis
Dialysis time, mon	7	3
Donor type	DCD	LD
HLA Mismatch, n	2(0-4)	1(0-4)
Kidney Transplantation Date	Aug 2005	Jan 2011
Previous transplantation	Yes	Yes
Symptoms of Fanconi syndrome	July 2016	Jan 2021
Main Symptoms	bone pain and scoliosis	increased creatinine
Date to Diagnose Fanconi syndrome	July 2017	Jan 2021
Operation to diagnosis	12 years	10 years
Medication, year	Adefovir dipivoxil 10mg Qd, 8	Tacrolimus 1mg Bid, 10

BMI, Body Mass Index; DCD, donation after cardiac death; LD, living donor; Qd, once a day; Bid, twice daily.

### Data collection

Relevant laboratory test results of the ADV and tacrolimus patients are presented in **Table 2**. Before the manifestation of symptoms related to Fanconi syndrome, the serum creatinine levels of the ADV and tacrolimus patients were 87 and 133 μmol/L, respectively, which were both within the normal range. The serum urea nitrogen and serum uric acid levels were normal. The electrolyte levels, including blood and urine phosphorus, potassium, and calcium, were normal. Neither patient had acidosis; their glucose metabolism was normal, and no β-2 microglobulin was detected in the urine, indicating normal renal tubular filtration function. On the day of diagnosis, the ADV patient had the following findings: serum creatinine, 247 μmol/L; blood urea nitrogen, 9.2 mmol/L; blood uric acid, 137 μmol/L; meanwhile, the tacrolimus patient had the following findings: serum creatinine, 490 μmol/L; blood urea nitrogen, 35.3 mmol/L; blood uric acid, 125 μmol/L. Renal dysfunction occurred in the two patients. At the same time, the two patients developed hypouricemia, which is one of the diagnostic criteria for Fanconi syndrome. In terms of renal tubular function, severe hypophosphatemia (0.48 and 0.46 mmol/L, respectively), severe hypokalemia (2.87 and 2.19 mmol/L, respectively), and hypocalcemia (2.15 and 1.16 mmol/L, respectively) occurred in the ADV and tacrolimus patients. In the detection of urine components, the ADV and tacrolimus patients developed hyperphosphatemia (88.3 and 90.3 mmol/24 h, respectively), hypercalciuria (13.35 and 20.9 mmol/24 h, respectively), glycosuria (3+ and 2+, respectively), and β-2 microglobulinuria (56.9 and 75 mg/L, respectively). Excessive loss of electrolytes results in various metabolic complications, and the ADV and tacrolimus patients also developed metabolic acidosis (20 and 9.6 mmol/L, respectively). The above series of laboratory tests confirmed the renal tubular function damage in the two patients, which supports the diagnosis of Fanconi syndrome. ADV and tacrolimus were immediately discontinued; thereafter, the biochemical indicators of the patients’ blood and urine gradually improved and renal function went back to normal. The tacrolimus patient, who was followed up for 500 days, had the following findings: serum creatinine, 129 μmol/L; blood urea nitrogen, 6.33 mmol/L; blood uric acid, 231 μmol/L; serum phosphorus, serum potassium, and serum calcium were all within the normal range. Urine phosphorus, potassium, and calcium were not detected. Glucose metabolism and bicarbonate were normal, and β-2 microglobulin was not detected. The ADV patient, who was followed up for 1825 days, had the following findings: serum creatinine, 145 μmol/L (slightly higher than normal); blood urea nitrogen, 5.9 mmol/L; blood uric acid, 330 μmol/L; blood and urine phosphorus, potassium, and calcium were all within the normal range. Glucose metabolism and bicarbonate were normal, and β-2 microglobulin was not detected.

**Table 2:** Laboratory Values in Relation to Presentation.

Laboratory Values in Relation to Presentation		Body Fluid	Creatinine,	Urea Nitrogen,	Uric Acid,	Phosphorus,	Potassium,	Calcium,	Bicarbonate	Glucose	B2-Micro Glycoprotein
			umol/L/-	mmol/L/-	umol/L/-	mmol/L/mmol/24h	mmol/Lmmol/24h	mmol/Lmmol/24h	mmol/L/-	-	-/mg/L
Symptoms of Fanconi syndrome before	Adefovir	Blood/Urine	87/-	6.9/-	337/-	0.97/30.28	4.5/67.8	1.98/1.7	24/-	N/N	-/-
	Tacrolimus		133/-	4.6/-	355.6/-	0.92/21.9	3.55/53.8	1.97/2.3	25/-	N/N	-/-
Day 0	Adefovir	Blood/Urine	247/-	9.2/-	137/-	0.48/88.3	2.87/70	2.15/13.35	20/-	N/3+	-/56.9
	Tacrolimus		490/-	35.3/-	125/-	0.46/90.3	2.19/81.1	1.16/20.9	9.6/-	N/2+	-/75
Day 2	Adefovir	Blood/Urine	223/-	8.8/-	133/-	0.49/79.2	3.1/65	2.2/13.33	18.8/-	N/2+	-/-
	Tacrolimus		438/-	33.9/-	147/-	0.51/78.9	2.77/82.9	1.4/21.34	11.3/-	N/1+	-/-
Day 7	Adefovir	Blood/Urine	148/-	6.6/-	145.5/-	0.73/50.2	3.33/53	2.23/12.45	18.9/-	N/N	-/52.1
	Tacrolimus		220/-	26.1/-	238.1/-	0.78/48.11	3.55/51.6	1.97/11.97	19.5/-	N/N	-/69.9
Day 18	Adefovir	Blood/Urine	143/-	6.7/-	277/-	1.1/19.8	3.9/45	2.39/8.32	23.1/-	N/2+	-/4.1
	Tacrolimus		155/-	10.3/-	329.5/-	1.13/17.3	3.9/31.1	2.33/12.19	24.2/-	N/N	-/13.5
Day 500	Adefovir	Blood/Urine	135/-	5.2/-	349/-	1.37/18.7	4.7/-	2.15/3.37	24/-	N/N	-/0.05
	Tacrolimus		129/-	6.33/-	231/-	1.33/-	4.11/-	2.28/-	25.7/-	N/N	-/N
Day 1825	Adefovir	Blood/Urine	145/-	5.9/-	330/-	1.23/17.9	4.83/32.5	2.37/1.9	23.4/-	N/N	-/-
	Tacrolimus		–	–	–	–	–	–	–	–	–
Reference Range		Blood/Urine	44~133/-	2.9~7.14/-	155~357/-	0.85~1.51/16~48	3.5~5.3/51~102	2.11~2.52/2~7.5	22~29/-	N	-/0~0.3

N, negative.

### eGFR trend before and after diagnosis of Fanconi

**Figure 2:**
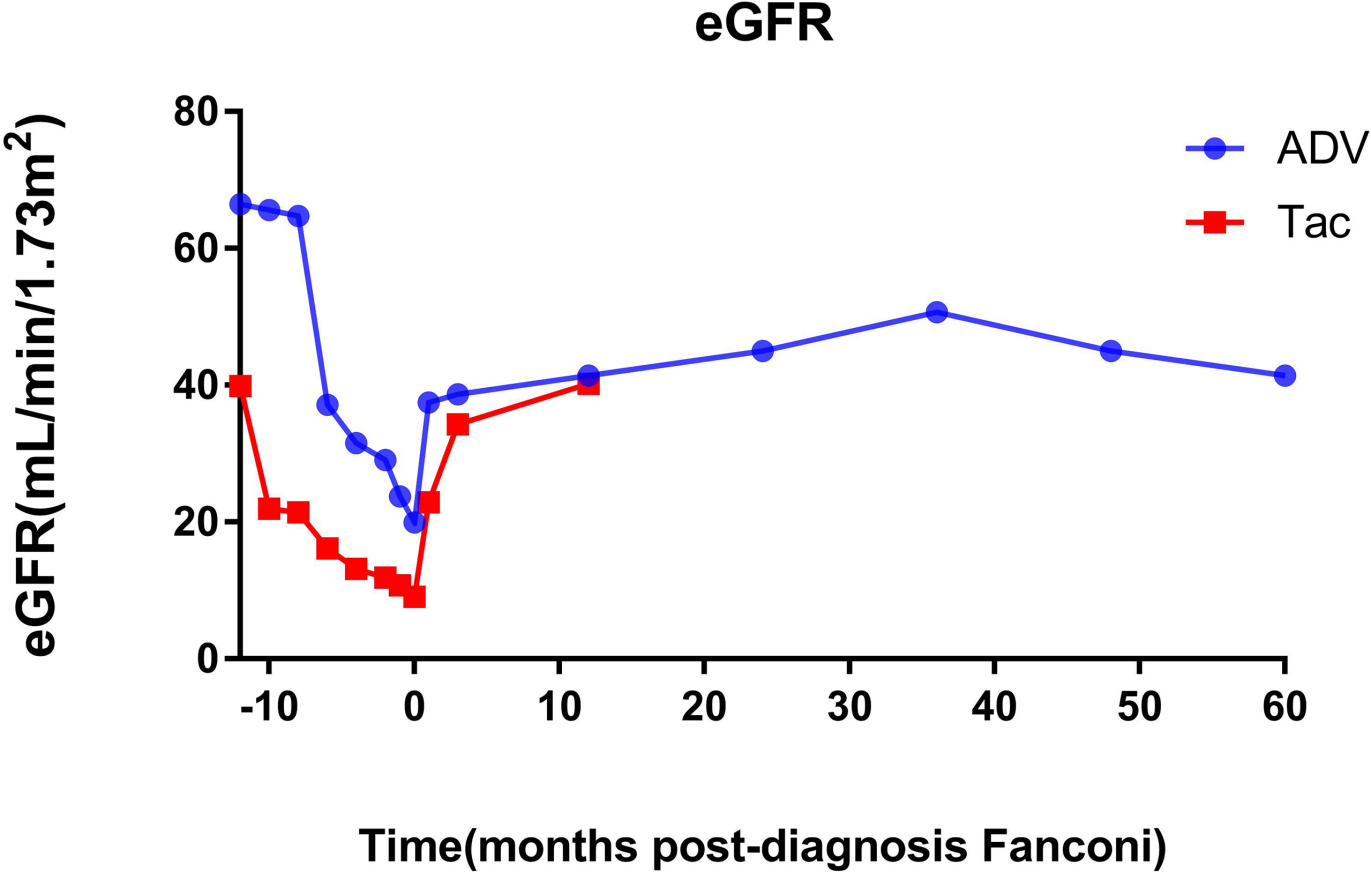
Changes of eGFR before and after diagnosis of Fanconi syndrome. eGFR, estimated glomerular filtration rate; ADV, adefovir dipivoxil; Tac, tacrolimus.

Before the diagnosis of Fanconi syndrome (>10 months), the eGFR of patients with ADV and tacrolimus remained at approximately 65 and 40 mL/min/1.73 m^2^, respectively. After kidney transplantation, only one kidney can function; thus, the eGFR was not too high, but it was relatively stable enough to maintain the needs of the recipients. However, due to the gradual aggravation of drug damage, the eGFR of the patients gradually decreased, and the ADV and tacrolimus patients reached their lowest eGFR (19.94 and 9.09 mL/min/1.73 m^2^) until the diagnosis of Fanconi syndrome. Even so, after immediate drug withdrawal and active symptomatic treatment, the eGFR of the two patients recovered significantly, and they were followed up for approximately 5 years (1825 days) and 1 year (500 days). The eGFR of the two patients increased to 41.45 and 40.25 mL/min/1.73 m^2^, respectively (**Figure 2**).

### Bone mineral density and T-score

**Figure 3:**
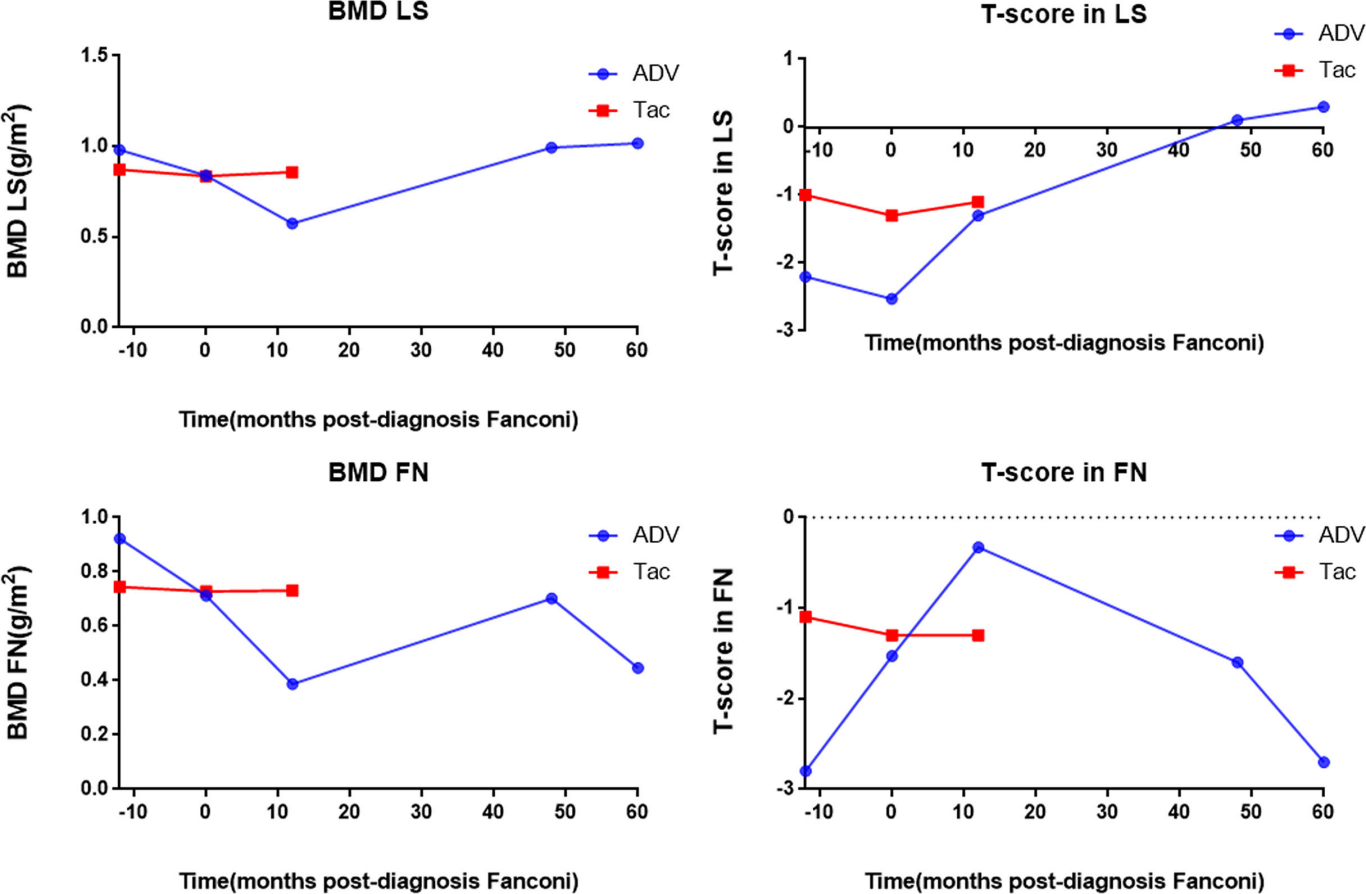
Bone mineral density and T-score of the patients. BMD LS, bone mineral density lumbar spine; T-score in LS, T-score in lumbar spine; BMD FN, bone mineral density femoral neck; T-score in FN; T-score in femoral neck, ADV, Adefovir dipivoxil; Tac, Tacrolimus.

**Figure 3** shows the bone mineral density and scores of the patients. The ADV patient had severe hypophosphatemic osteomalacia due to Fanconi syndrome, with accompanying bone destruction and osteoporosis. Dual-energy x-ray absorptiometry scans showed that the lumbar spine bone mineral density was 0.981 g/cm^2^ before the diagnosis of Fanconi syndrome, which reached the lowest level (0.574 g/cm^2^) >12 months after the diagnosis. It gradually recovered to 1.017 g/cm^2^ at the current follow-up at approximately 60 months after drug discontinuation. The bone mineral density score also changed from severe osteoporosis (−2.53) and gradually increased and reached a normal level (0.3). The bone mineral density of the femoral neck ranged from 0.922 g/cm^2^, before the diagnosis of Fanconi syndrome, to 0.387 g/cm^2^, at more than 12 months after the diagnosis and gradually recovered after drug withdrawal, and the bone mineral density was 0.446 g/cm^2^ at the most recent follow-up (approximately 60 months). The bone mineral density score was −2.53 in severe osteoporosis, and it was −2.7 at the current follow-up at 60 months. Although the bone mineral density of the femoral neck improved after drug withdrawal, severe osteoporosis remains during the long-term follow-up. Owing to the timely diagnosis of the tacrolimus patient, no changes in the lumbar spine or bone mineral density have occurred yet, and the bone mineral density and score were normal until follow-up at 12 months.

### Bone health-related laboratory parameters and imaging examination

**Figure 4:**
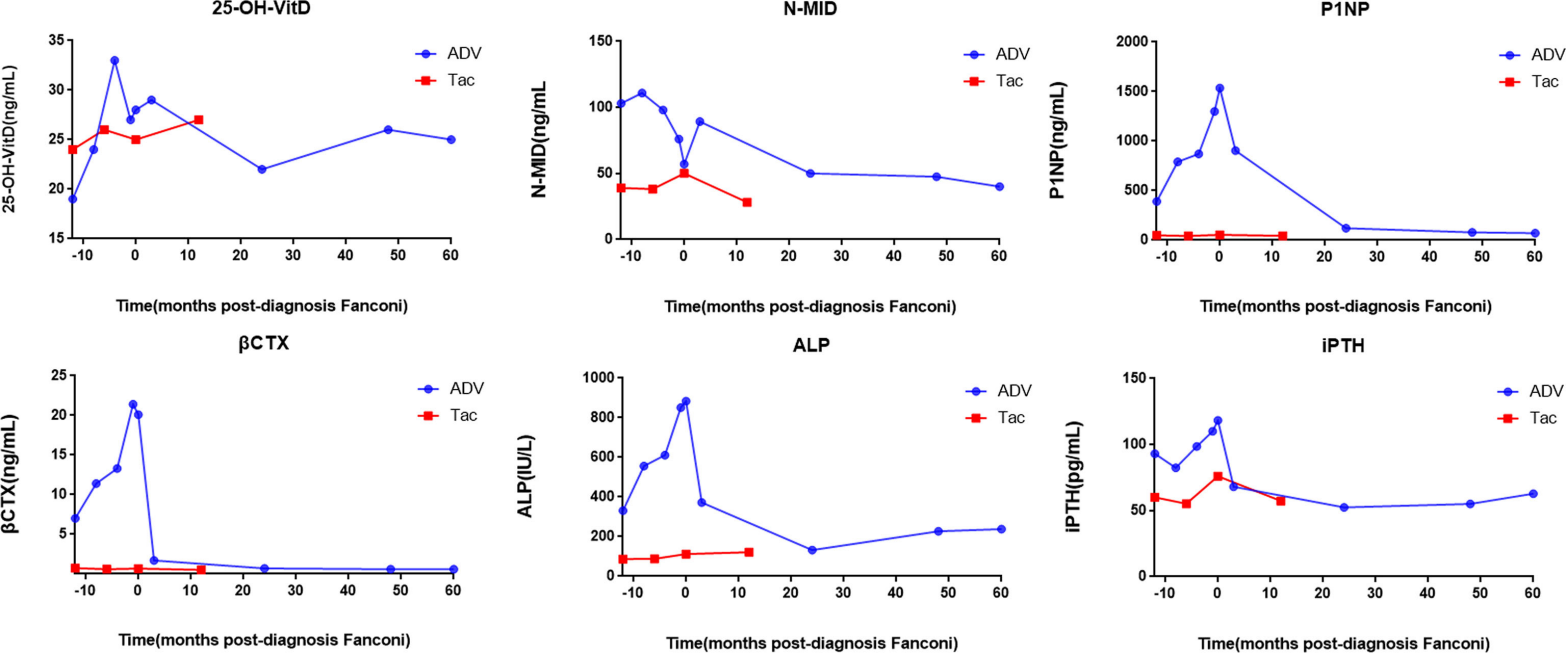
Changes of bone metabolism index before and after diagnosis of Fanconi syndrome. 25-OH-VitD, 25 hydroxyVitamin D; N-MID, N-terminal mid-fragment; P1NP, procollagen type 1 N-terminal peptide; βCTX, β-isomerized cross-liked C-telopeptide of type 1 collagen; ALP, alkaline phosphatase; iPTH, intact parathormone.

**Figure 5:**
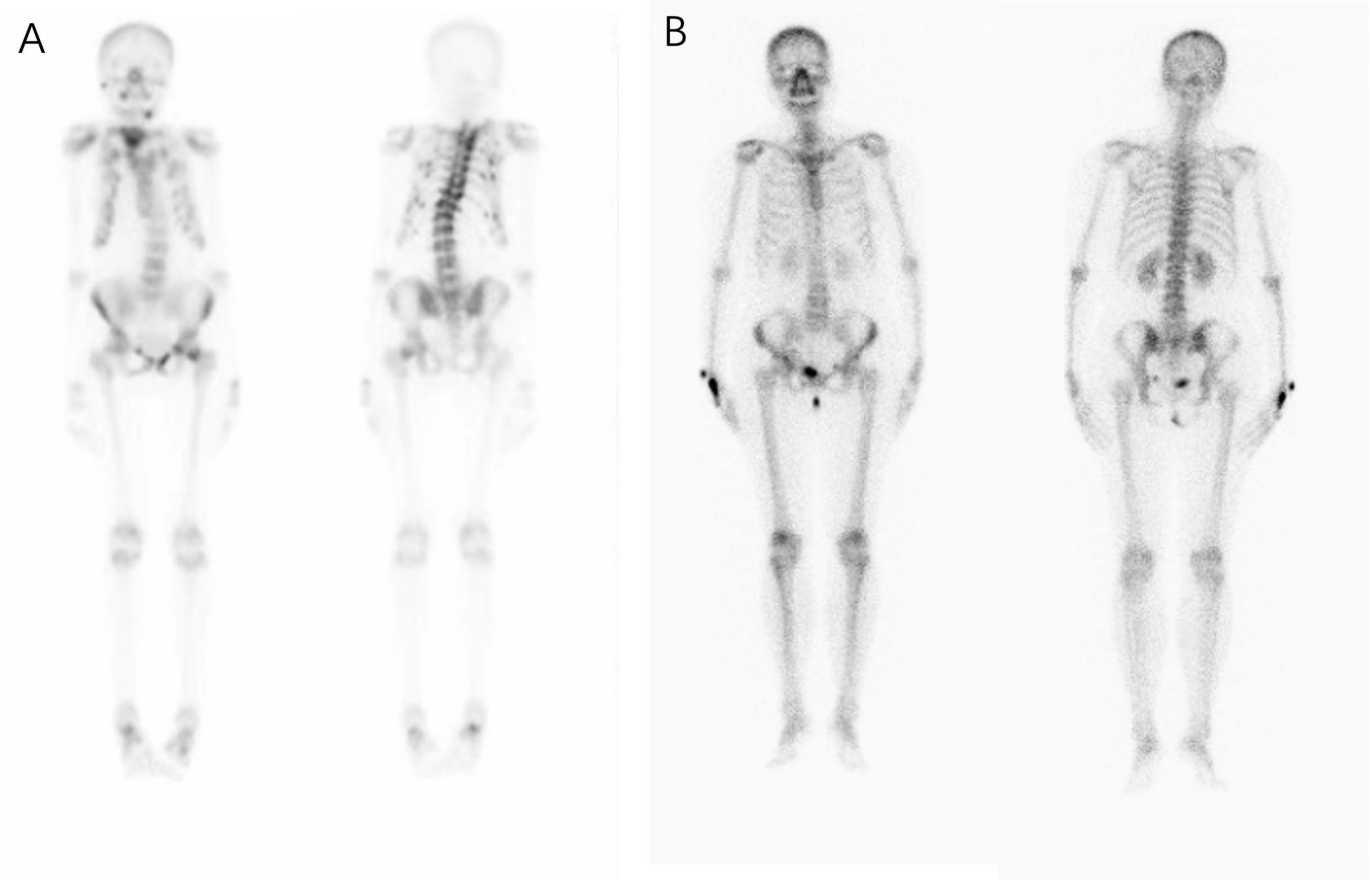
Whole body bone scan of the patients. Complete single-photon emission computed tomography whole-body bone scan at the onset of the disease detecting an increased uptake of radioactive tracers and possible osteomalacia changes in bone lesions. **(A)** In patients who were taking adefovir dipivoxil, scoliosis was obvious, multiple vertebral bodies became flattened, multiple vertebral bodies and multiple ribs were abnormally distributed and concentrated, and local bone metabolism was active. **(B)** No obvious abnormal radioactive distribution or aggregation was found in the whole-body bone scan of the patient taking tacrolimus.

**Figure 6:**
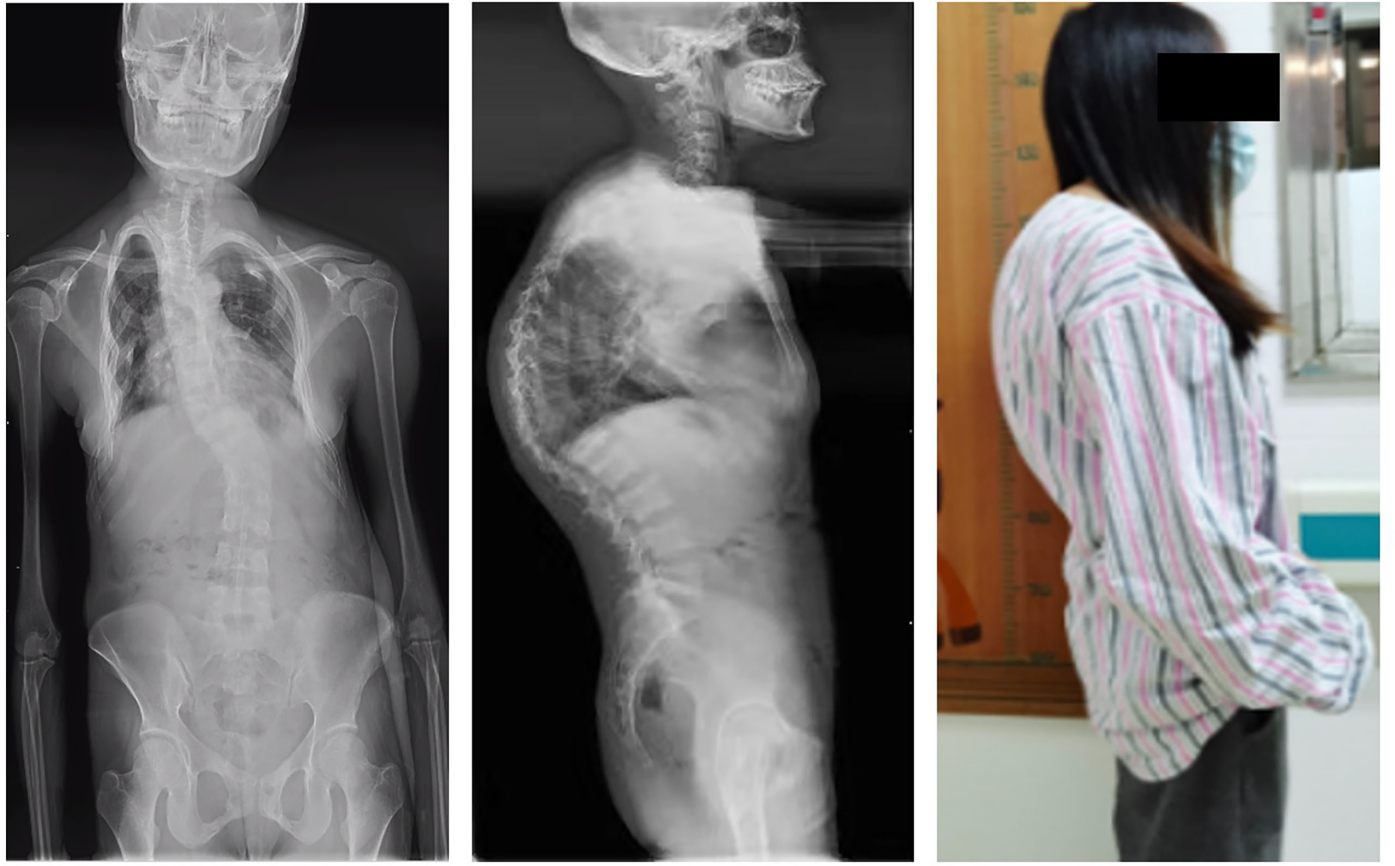
Whole-spine splicing X-ray anterior and lateral views. At the onset of the disease, the adefovir patient obvious severe scoliosis: rightward bending deformity.

The patients’ laboratory tests of bone health-related metabolism before the diagnosis of Fanconi syndrome (>10 months), at the diagnosis of Fanconi syndrome, and during follow-up are presented in **Figure 4**. The main clinical manifestations of the ADV patient were bone pain and osteomalacia, and the destruction of bone was subsequently observed during bone metabolism examination. The N-MID gradually decreased after onset and was the lowest at the time of diagnosis, whereas P-1NP, βCTX, ALP, and iPTH gradually increased after the onset, reaching the peak at the time of diagnosis. At that point, the bone destruction was the most serious, and the patient also developed severe osteomalacia. There were multiple abnormal bone radioactive distribution and aggregation foci, local active bone metabolism (**Figure 5A**), and obvious severe scoliosis on whole-body bone scan (**Figure 6**),the ADV patient’s height decreased to 132.6 cm. After immediate drug discontinuation and symptomatic treatment, the patient’s P-1NP, βCTX, ALP, and iPTH level gradually decreased and were maintained at a low level, but N-MID remained unchanged. 25-OH-VitD remained at a relatively normal level due to the consideration of osteoporosis treatment and supplementation of exogenous vitamin D after the onset of the disease. The height of the patient was 138 cm, which was slightly higher than the previous 132.6 cm during 5-year follow-up. As the bone was not destroyed in the tacrolimus patient, only renal function was damaged, and the bone metabolism level was at a normal level before and after diagnosis, with no significant change, and no obvious abnormality was found in the whole-body bone scan (**Figure 5B**).

### Microscopy report of renal allograft biopsy

**Figure 7:**
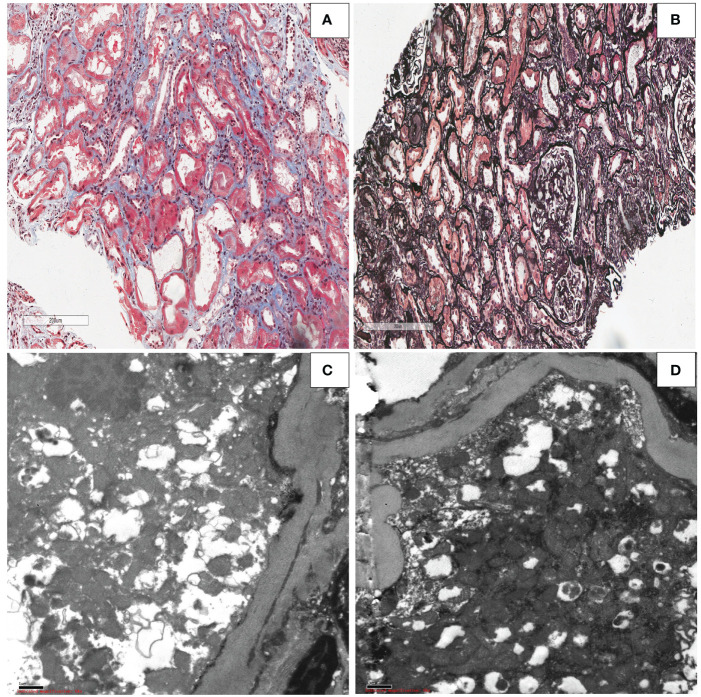
Kidney allograft biopsy findings in the patient with Fanconi syndrome who was taking adefovir dipivoxil.**(A)** Light microscopy showing shedding of the epithelial cells of the renal tubules, exposed basement membrane, regeneration of some renal tubular epithelial cells, red blood cells in the lumen, and scattered inflammatory cells in the interstitium (Masson, original magnification ×50). **(B)** Light microscopy showing renal tubular atrophy and interstitial fibrosis, renal vascular thickening, hyaline degeneration, and glomerular segmental sclerosis (periodic acid–Schiff, original magnification ×50). **(C, D)** Electron microscopy showing swelling,deformity and morphological disorder of mitochondria in renal tubular epithelial cells (electron micrograph, original magnification, ×1000).

The transplanted kidney biopsy of the ADV patient revealed the following light microscopy results: shedding of renal tubular epithelial, exposed basement membrane, regeneration of some renal tubular epithelial cells, red blood cells in the lumen, and scattered inflammatory cells in the interstitium(**Figure 7A**). Renal tubular atrophy, interstitial fibrosis, renal vascular thickening, hyaline degeneration, and glomerular segmental sclerosis were also observed (**Figure 7B**). Electron microscopy results showed vacuolar degeneration of renal tubular epithelial cells, partial dilation of cavities, shedding of epithelial villi, and swelling and deformity of the mitochondria in renal tubular epithelial cells. Renal interstitial edema with lymphoid and mononuclear cell infiltration was also observed (**Figures 7C, D**). These changes, provided the histological basis for the diagnosis of Fanconi syndrome.

## Discussion

To our knowledge, this is the first report on the clinical manifestations, test results, diagnostic process, treatment, and prognosis of drug-induced Fanconi syndrome after kidney transplantation. The secondary osteomalacia symptoms of Fanconi syndrome caused by adefovir can be misdiagnosed as glucocorticoid-induced osteoporosis after kidney transplantation. Fanconi syndrome secondary to tacrolimus, which is the most commonly used immunosuppressant after kidney transplantation, is difficult to diagnose. However, through rigorous and effective clinical thinking for diagnosis and timely discontinuation of the drugs that cause Fanconi syndrome, a relatively satisfactory curative effect can be achieved.

Primary Fanconi syndrome generally refers to idiopathic Fanconi syndrome without obvious triggers. The incidence rate after kidney transplantation has not been reported. The incidence of idiopathic Fanconi syndrome in kidney transplant patients at our center is 1 in 1728 cases. Secondary Fanconi syndrome includes two categories, one is hereditary and the other is acquired. Hereditary factors include cystinosis, galactosemia, hereditary fructose intolerance, glycogenopathy, hereditary tyrosinemia type I, Wilson disease, Row syndrome, Dante disease, and mitochondrial cytopathic. Acquired factors are mainly heavy metal poisoning (such as lead and cadmium), chemotherapy drugs (such as cisplatin and cyclophosphamide), anti-nucleotide drugs (such as ADV and tenofovir), valproate, and Chinese herbal medicines containing *Aristolochia*, and Fanconi syndrome secondary to these drugs has been reported (17–21). In particular, Fanconi syndrome secondary to ADV is not uncommonly reported in patients with chronic viral hepatitis B (22). There are also some drugs that may cause Fanconi syndrome that have not yet been reported. No case of Fanconi syndrome caused by tacrolimus has been reported until now. In nearly 3000 cases of kidney transplantation in our center, no other calcineurin inhibitor, except for tacrolimus, has been found to cause Fanconi syndrome. The incidence is low, but once it occurs, it can cause a series of serious clinical signs and symptoms. If the drug is not discontinued and symptomatic treatment is not timely, it may be life-threatening. Because of the dysfunction, a large amount of glucose, amino acids, phosphates, etc. are eliminated in the urine, manifesting as renal glycosuria, aminoaciduria, phosphateuria (hypophosphatemia), uricuria (hypouricemia), proximal renal tubules acidosis, etc. The disease is mainly clinically manifested as systemic skeletal pain, osteomalacia, severe skeletal deformities, and a group of syndromes, including hypokalemic paralysis, fatigue, and growth retardation in children, which can lead to systemic multisystem damage and can even be life-threatening (17).

In our study, the ADV patient was found to be positive for HBsAg after kidney transplantation; thus, long-term anti-HBV treatment (oral ADV, 10 mg, once a day), was given, which is very common in kidney transplant patients. Chronic viral hepatitis B is a disease characterized by chronic liver inflammation caused by persistent HBV infection. The low immunity after kidney transplantation can easily cause explosive hepatitis. Long-term anti-HBV drug treatment is needed to control the HBV-DNA at a low level. More than 350 million people worldwide are HBV carriers. China is a high-incidence area of HBV infection. Currently, there are approximately 93 million cases of chronic HBV infections, accounting for almost one-third of worldwide HBV cases (23–25). Drugs used for the treatment of chronic hepatitis B have two types: interferon and nucleoside/nucleotide analogues. However, due to the obvious side effects of interferon and the low rate of treatment compliance, its clinical use is limited. Nucleotide/nucleotide analogues are the main drug for the treatment of hepatitis B. ADV is a nucleotide virus reverse transcriptase inhibitor with broad-spectrum antiviral activity. It was approved by the US Food and Drug Administration (FDA) in September 2002 for the treatment of chronic hepatitis B and was launched in China in April 2005. In China, ADV is recommended as the first-line anti-HBV drug for adult chronic hepatitis B patients with naïve or lamivudine resistance. The incidence of Fanconi syndrome secondary to nucleotide drugs (especially ADV) has gradually increased in recent years (26). Hypophosphatemia and bone pain symptoms may appear after long-term medication for many years and gradually aggravate and develop into osteomalacia, which can cause irreversible skeletal deformities. Previous study showed that the pathogenesis of Fanconi syndrome caused by long-term administration of low-dose ADV may be related to two transporters, OAT1 and MRP2. OAT1 overexpression and MRP2 inhibition lead to ADV accumulation in proximal renal tubular cells (27, 28).

Osteopenia and osteoporosis are the common complications after kidney transplantation. At the same time, kidney transplant recipients are most susceptible to bone diseases due to various chronic kidney diseases before surgery, which manifest as hyperphosphatemia, hypocalcemia, increased parathyroid hormone, abnormal vitamin D levels, increased alkaline phosphatase levels, and metabolic acidosis. Although kidney transplantation improves the recipient’s renal function and quality of life, abnormal bone metabolism persists. More than 15% of kidney transplant patients develop osteoporosis in the first year after transplantation (29), and the risk of fracture is also increased (30, 31). Nikkei et al. (30) conducted a statistical analysis of fracture events in 68,814 kidney transplant recipients and found that approximately 22.5% of kidney transplant recipients had fractures within 5 years after kidney transplantation, and the risk was approximately 4 times that of the general population. Glucocorticoids have the most adverse effects on bone microarchitecture, especially in the early post-renal transplant patients period, the use of high-dose glucocorticoids is associated with rapid bone loss and high fracture rates. Glucocorticoids impair intestinal calcium absorption by inhibiting the action of vitamin D, increasing renal calcium loss, and affecting the conversion of calcidiol to calcitriol to reduce calcium concentration, thereby increasing calcium consumption and reducing calcium absorption. It also stimulates parathyroid chief cells to secrete PTH, thereby inducing hyperparathyroidism. Studies have shown that glucocorticoids also impair the synthesis of insulin-like growth factor 1 and gonadal function. Impaired gonadal function can lead to decreased secretion of gonadal hormones, stimulated osteoclasts, and inhibited osteoblast differentiation. Increased apoptosis of osteoblasts and osteocytes aggravates bone loss (32, 33). Osteoporosis caused by the hormone use is well known to transplant surgeons.

However, post-renal transplant patients who take glucocorticoids for a long time may have accompanying chronic viral hepatitis that requires long-term ADV treatment. Osteomalacia and skeletal deformities are not yet present in the early stage of the disease. At this stage, bone pain and bone loss are the main symptoms, which are easily confused with osteoporosis caused by hormones, leading to misdiagnosis (34). In this study, the patient with long-term ADV intake (10 mg, once a day) was 160 cm tall before the symptoms appeared. Bone pain began to appear after the 11th year of taking the drug. When she was treated in the hospital, the examination results of the patient were consistent with osteoporosis, and there was no change in renal function damage or proximal renal tubular damage. The doctor prescribed vitamin D, calcium, and calcitriol for anti-osteoporosis treatment. However, the patient’s bone pain did not improve significantly; furthermore, it aggravated and mild scoliosis appeared. Eventually, there was manifestation of Fanconi syndrome osteomalacia; however, the patient was misdiagnosed with glucocorticoid-induced severe osteoporosis. In the follow-up treatment, because of the continuous aggravation of scoliosis, bisphosphonates were added to induce osteoclast apoptosis, inhibit bone resorption, and improve bone mineral densit (35). The patient’s pain improved, but the scoliosis continued to aggravate. The damage to the tubules gradually became obvious. After 1 year with the disease, the serum creatinine also continued to increase. Transplanted kidney biopsy was performed,swelling and deformity of the mitochondria in renal tubular epithelial cells. These are typical changes occurring with renal tubular damage and an important basis for Fanconi syndrome diagnosis (36). After 12 years of taking the drug, the patient was diagnosed with Fanconi syndrome secondary to ADV. After drug withdrawal and symptomatic and supportive treatment, the patient’s renal function and proximal renal tubular damage gradually improved. Timely detection and active treatment of Fanconi syndrome can lead to good results (34, 37). However, severe scoliosis is difficult to reverse, which has a serious impact on the quality of life of the patient. On the 5th year after the patient’s diagnosis of Fanconi syndrome, the height of the patient was 138 cm, which was slightly higher than the previous 132.6 cm. We believe that this was due to a slight recovery of the scoliosis after the improvement of osteomalacia.

Tacrolimus is a calcineurin inhibitor, which is a macrolide immunosuppressant extracted from the fermentation broth of Streptomyces tsukubaensis. It was approved by the FDA in 1994 for solid-organ transplantation. At present, it is widely used in the prevention and treatment of rejection after various organ transplantations, which is an important part of the immunosuppressive regimen after kidney transplantation (38). Relevant data showed significant differences in the efficacy of tacrolimus in different populations (39, 40). Although therapeutic drug monitoring is routinely performed in clinical practice, some drug-related adverse reactions are still unavoidable. Nephrotoxicity is an adverse reaction that affects patient prognosis (13, 41). The nephrotoxicity of changes in renal vascular resistance, which is reversible and manifested as decreased renal blood flow and decreased glomerular filtration rate. The chronic nephrotoxicity is mainly manifested as irreversible changes in renal structure, including renal vascular thickening and sclerosis, tubular atrophy, tubulointerstitial fibrosis, glomerulosclerosis, etc. Reducing the nephrotoxicity of tacrolimus is a major clinical problem that should be addressed urgently (14–16). The pathogenesis of tacrolimus nephrotoxicity in kidney transplant recipients is still being studied. Studies have shown that gene polymorphism is closely related to tacrolimus drug concentration (42); therefore, detecting the nephrotoxicity of tacrolimus and other diseases caused by it, such as Fanconi syndrome, is very important.

In this study, the patient regularly took tacrolimus (1 mg, twice a day) for a long time after kidney transplantation, and the tacrolimus trough concentration was regularly monitored to be no less than 6 ng/mL. Except for long-term combined use of mycophenolate mofetil and methylprednisolone to maintain immunity, no other drugs were taken, and there was no occurrence of heavy metal poisoning. Ten years after kidney transplantation, the patient had an increase in creatinine, which may be initially considered as nephrotoxicity caused by tacrolimus. We adjusted the dosage of the drug to reduce the trough concentration of tacrolimus, but the patient’s creatinine gradually increased from normal to 490 μmol/L, and fatigue occurred. There were no other specific manifestations, and the urine output did not decrease. The population-reactive antibodies showed negative results, and other related antibody investigations showed no abnormality. The ultrasound Doppler of the transplanted kidney showed that the blood flow of the transplanted kidney was abundant, and the renal vascular resistance index was not high. Based on the findings, the possibility of rejection and prerenal damage was not considered. We found that the patient had obvious evidence of proximal tubular injury, increased serum creatinine, and azotemia, but the serum uric acid was significantly decreased, and the patient developed severe hypophosphatemia, severe hypokalemia, severe hypocalcemia, severe metabolic acidosis, and urine β-2 microglobulin, which met the diagnostic criteria for Fanconi syndrome (17). The tacrolimus was immediately discontinued, and cyclosporine A was used to continue maintenance immunotherapy. After tacrolimus discontinuation, phosphorus, potassium, and calcium supplementation, and acidosis correction, the patient’s serum creatinine decreased to 220 μmol/L on the 7th day, serum phosphorus and calcium were close to normal, serum potassium increased and was completely normal, and urine glucose also disappeared. Owing to the detection and diagnosis of the disease in the early stage, the patient’s bone mineral density, whole-body bone scan, and bone metabolism indexes were normal. Since the patient’s renal function gradually returned to normal, invasive examinations such as transplanted kidney biopsy were not performed.

This study focused on the characteristics of Fanconi syndrome after kidney transplantation, which can reduce incorrect and missed diagnosis and thus benefit transplant patients. The limitations of our study is that number of patients is limited and tacrolimus patient have no kidney allograft biopsy. Exploring potential spectific biomarkers to identify Fanconi syndrome in the early stage deserves further research.

## Data availability statement

The raw data supporting the conclusions of this article will be made available by the authors, without undue reservation.

## Ethics statement

The studies involving human participants were reviewed and approved by Ethics Committee of the Second Xiangya Hospital of Central South University. The patients/participants provided their written informed consent to participate in this study. Written informed consent was obtained from the individual(s) for the publication of any potentially identifiable images or data included in this article.

## Author contributions

ZT, TL and HD drafted the manuscript. ZT, CHF, MN, XY, YF, JP, SS, MZ and CF collected data. JP and MZ generated the figures. XX, HZ, FP, GL, SY, YW and XT performed the surgery. XZ and XJ made and analyzed the biopsies. LP, HD, ZT and TL designed the outline of the manuscript and revised the manuscript. All authors contributed to the article and approved the submitted version.

## Funding

This work is supported by the National Science Foundation of China (82070776, 81900370 and 81970655), Excellent Youth Foundation of Hunan Province of China (2021JJ10076) and Huxiang Young Talents of Hunan Province (2019RS2013).

## Conflict of interest

The authors declare that the research was conducted in the absence of any commercial or financial relationships that could be construed as a potential conflict of interest.

## Publisher’s note

All claims expressed in this article are solely those of the authors and do not necessarily represent those of their affiliated organizations, or those of the publisher, the editors and the reviewers. Any product that may be evaluated in this article, or claim that may be made by its manufacturer, is not guaranteed or endorsed by the publisher.
